# Restrictive Lung Pattern: A Rare Complication of a Phytobezoar

**DOI:** 10.1097/PG9.0000000000000056

**Published:** 2021-03-08

**Authors:** Morgan Doughty, Jane Alookaran, Marc Rhoads, Paul H. Dahm

**Affiliations:** From the * Department of Pediatrics, Division of Critical Care Medicine, McGovern Medical School, part of UTHealth, The University of Texas Health Science Center at Houston, Houston, TX; and; † Department of Pediatrics, Division of Gastroenterology, McGovern Medical School, part of UTHealth, The University of Texas Health Science Center at Houston, Houston, TX.

## INTRODUCTION

Bezoar is an “umbrella term” that describes collections of indigestible material that becomes trapped anywhere within the gastrointestinal system. Bezoars can be further classified according to their components: pharmacobezoars (collections of medications), trichobezoars (collections of hair), phytobezoar (collections of plant material), and lactobezoar (collections of coagulated milk protein) (1). Patients at risk for the development of bezoars are typically those with underlying neurological, psychiatric, or developmental disorders. Other risk factors include diets high in fiber, poor fluid intake, immobility, previous gastric surgeries, and gastrointestinal dysmotility (2).

The diagnosis of a bezoar requires a high index of suspicion, as some patients may not be able to adequately verbalize their symptoms, and the symptoms may be nonspecific. The most common symptoms include nausea, vomiting, and abdominal pain. The most common complications include ulceration, anemia, obstruction, or perforation of the gastrointestinal tract (3). More severe complications include weight loss, loss of absorption, anemia secondary to bleeding, and small intestinal obstruction and/or perforation (4).

The therapeutic modality of choice for bezoars is esophagogastroduodenoscopy (EGD). The advantages of endoscopy are the ability to visualize the bezoar, the capability to sample the contents, and the ability to treat by removal (5, 6). Less invasive treatment methods such as with enzymatic or cola dissolution have also been employed with variable rates of success (7). We describe an 11-year-old male with an intellectual disability and seizure disorder who was found to have a phytobezoar after becoming severely hypoxemic.

Restrictive lung disease is characterized by decreased lung volumes. Varying causes of restrictive lung disease are commonly seen in the intensive care setting and can be divided into 2 categories: pulmonary and extrapulmonary. Pulmonary causes include interstitial lung diseases such as sarcoidosis or pulmonary fibrosis. Extrapulmonary causes include poor muscular effort as seen in myasthenia gravis or poor structural apparatus such as scoliosis (8). We present a unique case that describes a serious pulmonary-gastrointestinal complication.

## CASE REPORT

An 11-year-old boy with a medical history significant for intellectual disability and a seizure disorder was admitted to the pediatric intensive care unit after being found unresponsive in a bathtub after receiving 40 minutes of cardiopulmonary resuscitation. From family history, the patient’s seizures had been well-controlled on levetiracetam. Notably, he had also been seen by his primary care physician for chronic, recurrent bouts of nausea and emesis, currently managed with ondansetron. He had not received any further gastrointestinal workup and did not have any unusual dietary patterns.

**FIGURE 1.**
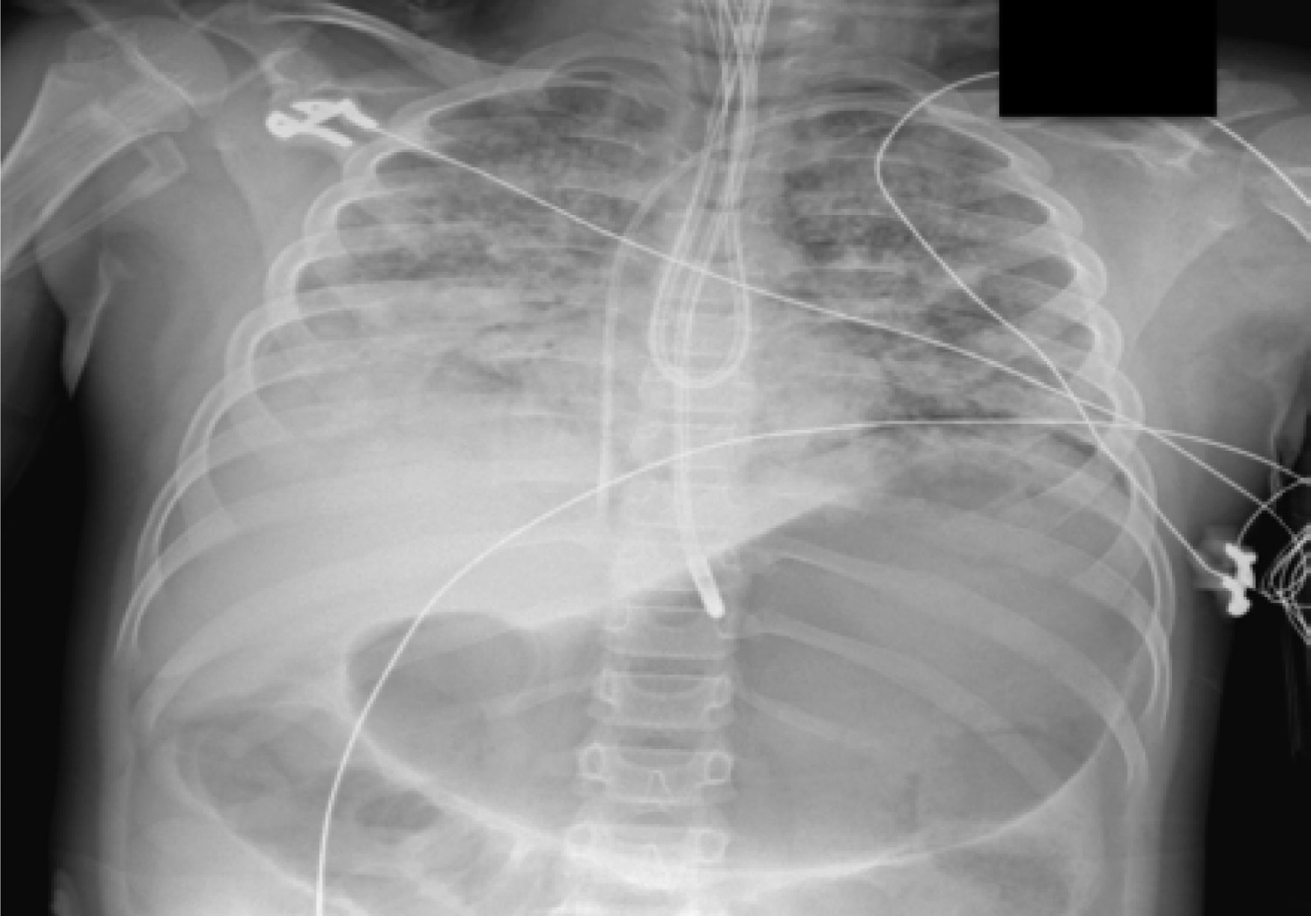
Chest and abdominal x-ray with the nasogastric tube unable to be advanced past the distal esophagus with hyperinflated gastric bubble.

The patient was initially ventilated with a bag valve mask and subsequently intubated and ventilated with an initial mean airway pressure (P_aw_) of 11 at the scene. His abdomen was noted to be soft at the scene. Upon arrival to the pediatric intensive care unit, the patient was noted to have a massively distended, noncompressible abdomen. His oxygen saturations were in the low 70’s while receiving fraction of inspired oxygen of 100% on conventional ventilation. Given no improvement with maximization of settings, he was converted to airway pressure release ventilation with minimal improvement in oxygen saturation. P_aw_ increased to the 50’s, at which time he was transitioned to high-frequency oscillatory ventilation. During this process, inhaled nitric oxide was started with slight improvement. Placement of a large-bore nasogastric tube (NGT) for decompression of the stomach was attempted multiple times. On chest and abdominal x-ray imaging, the NGT was repeatedly in the distal esophagus (Fig. 1). Given no improvement in hypoxemia on high-frequency oscillator ventilation with a P_aw_ of 48, pediatric gastroenterology was consulted for emergent EGD to determine the cause of obstruction in the distal esophagus.

**FIGURE 2.**
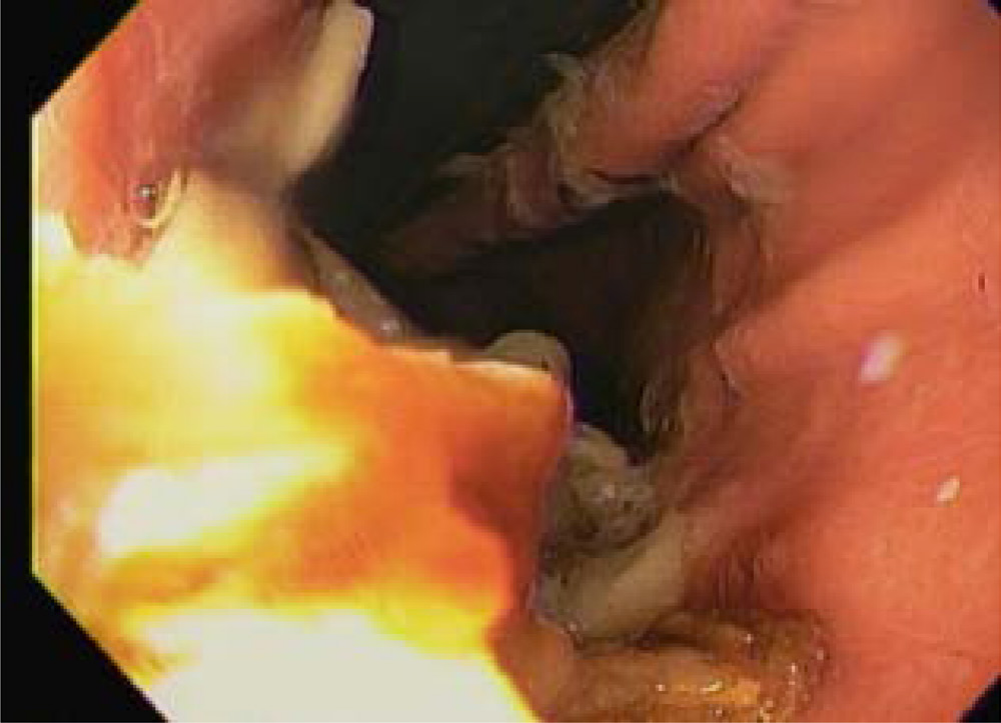
Initial visualization of the distal gastroesophageal junction partially occluded by the bezoar.

**FIGURE 3.**
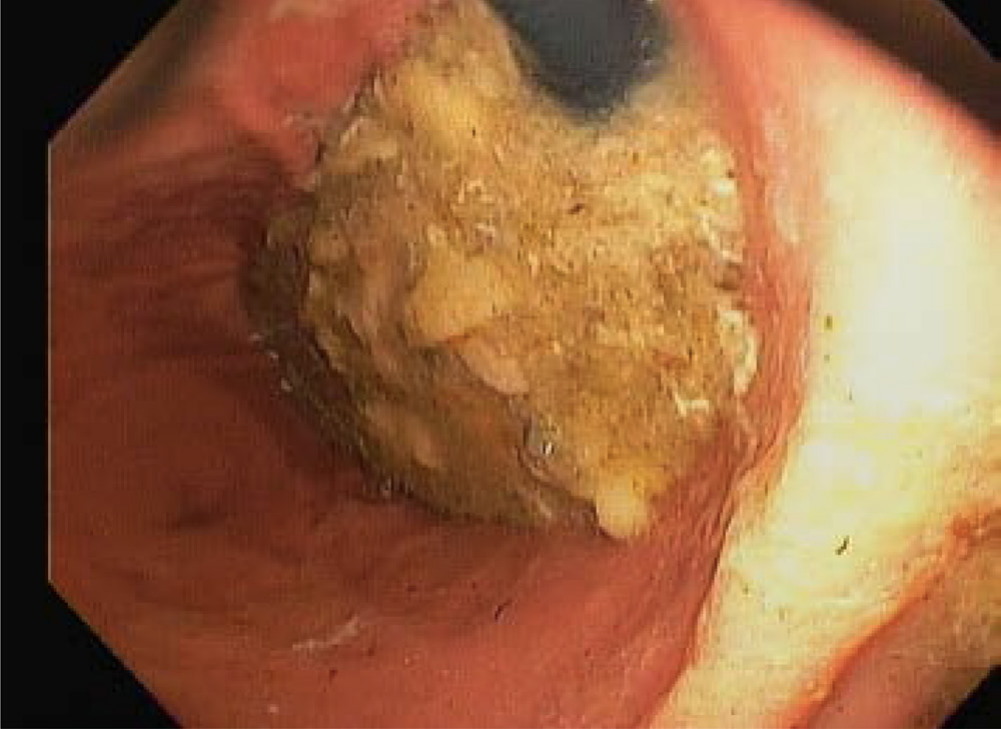
Retroflexed endoscopic view of the gastric cardia after partial bezoar suction removal, demonstrating a portion of the bezoar causing the ball valve effect, which led to the restrictive lung component.

An EGD was performed with an Olympus GIF-H190 with an outer diameter of 9.2 mm. EGD demonstrated the partial occlusion of the gastroesophageal junction by a phytobezoar (Fig. 2). The scope easily passed into the stomach. Upon entering the stomach, there was a loud release of air, and the patient’s oxygen saturation immediately increased to 100%. Advancing to the body of the stomach revealed a large phytobezoar in the stomach (Fig. 3). The bezoar was difficult to break up, requiring several instances of water irrigation and removal with a Roth net. Over 1 L of material was lavaged and suctioned out. No medications were visible in the extracted bezoar. His abdomen remained soft and compressible, and he was successfully transitioned back to conventional ventilation with P_aw_ in the teens.

## OUTCOME AND FOLLOW-UP

The unfortunate boy had sustained a severe anoxic brain injury secondary to his submersion injury and subsequently progressed to brain death.

## DISCUSSION

The diagnosis of a bezoar requires a high index of suspicion, given the nonspecific nature and wide spectrum of symptoms. In addition, many patients with bezoars have other underlying disorders, such as developmental disorders, which may preclude them from being able to accurately describe their symptoms. While most symptoms of bezoars are insidious in nature, our case report demonstrates a life-threatening bezoar complication in a critically ill patient.

We believe the patient’s initial injury to be secondary to a seizure and subsequent submersion injury with aspiration. However, he did not respond to bag-valve-mask ventilation upon initial resuscitation. It is believed that the bezoar at the gastroesophageal junction created a ball valve effect in which air entered his stomach but could not escape. This correlated clinically by his distended, noncompressible abdomen, which pushed up on his diaphragm decreasing the lung volume and hence creating a restrictive lung pattern and hypoxemia. The diagnosis of gastric bezoar was delayed given his mechanism of injury and infiltrates on chest x-ray. An extensive literature review did not yield any other reports of this unusual complication of the condition. Viveiros et al (9) reported a rare complication of left atrial compression from an esophageal bezoar consisting of diaper fragments as well as aspiration of foreign material presenting with cardiopulmonary complications.

Another important consideration, in this case, is the possibility that the onset of status epilepticus was related to the bezoar chronically preventing absorption of medications. Prevention of absorption could have occurred in 2 ways; either through the physical obstruction of the bezoar in the stomach delaying passage of medications to the small intestine or to frequent vomiting causing subtherapeutic antiepileptic levels. This patient had previously been well-controlled on an oral antiepileptic for his seizure disorder, and there were reports of good compliance. Antiepileptic levels were not obtained during his hospitalization, and therefore, medication compliance was based solely on history from family. In addition, history from his family was otherwise unremarkable for other causes of a lowered seizure threshold including recent illness or recent medication changes.

While EGD is both diagnostic and therapeutic for bezoars, other treatment modalities have been successfully used. The type of bezoar may dictate which therapeutic modality will be most successful. For example, trichobezoars often require surgical removal. Different modalities can be used in conjunction with endoscopy to help resolve bezoars including lithotripsy and enzymatic dissolution. Park et al (6) reported that surgical treatment of bezoars is occasionally indicated when there are complications such as intestinal perforation or removal of bezoar is technically challenging with EGD. Lastly, there are several case reports of using less invasive means of resolution of bezoars as seen with cola dissolution through a NGT (7, 10, 11).

In summary, the inability to place a NGT in a patient with progressively worsening ability to achieve adequate ventilation with an enlarged stomach should raise the concern about rare etiologies including gastric bezoar, and one should consider emergent endoscopy to evaluate.
